# Familiarity in the Context of Risk Assessment of Transgenic Crops: Focus on Some Countries in the Americas

**DOI:** 10.3389/fbioe.2019.00463

**Published:** 2020-01-28

**Authors:** Deise M. F. Capalbo, Phil Macdonald, Patricia Machado Bueno Fernandes, Clara Rubinstein, Carmen Vicién

**Affiliations:** ^1^Embrapa Environment and International Life Sciences Institute, São Paulo, Brazil; ^2^Canadian Food Inspection Agency, Ottawa, ON, Canada; ^3^Federal University of Espírito Santo, Vitoria, Brazil; ^4^Bayer Crop Science and International Life Sciences Institute, Buenos Aires, Argentina; ^5^University of Buenos Aires and International Life Sciences Institute, Buenos Aires, Argentina

**Keywords:** familiarity, history of safe use, risk assessment, problem formulation, regulatory framework, harmonization

## Abstract

Problem formulation is the formal opening stage of a risk assessment that determines its purpose and scope and hence guides the gathering of information data. The concepts of familiarity and history of safe use are an integral part of problem formulation. These concepts do not replace the case-by-case approach and are not taken as safety standards but are valuable components of the process that shape the generation of plausible, testable risk hypotheses. The International Life Sciences Institutes in Brazil and Argentina have facilitated numerous discussions on the scientific principles for risk assessment of transgenic crops in the Latin American region in the past 5–6 years. The session held at ISBR 15th elaborated on the familiarity concept and derived tools and their role in the evolution of risk evaluation criteria. Examples of how different countries in the Americas interpret and apply these conceptual tools show that familiarity is a valuable concept, although terms are very often confused and vaguely defined. Formalizing these terms with clear definitions and scope of application in guidelines and regulatory documents would reduce ambiguity, enhance predictability, and add transparency to the evaluation processes.

## Introduction

Risk assessment criteria for transgenic organisms have been set decades ago and are still current, built on the following: case-by-case, comparative assessment, tiered approach, and consideration of the weight of evidence. However, as science moves forward, new developments and knowledge make it necessary to periodically update and/or adjust these criteria (Borges et al., 2018).

Problem formulation has been defined as the “formal, structured, opening stage” of the risk assessment (Patton, 1998). It was originally described in the Environmental Protection Agency’s Framework Report (Norton et al., 1992; EPA, 2014) as a conceptual model that considers the values to be protected, the data needed, and the analyses to be used. Problem formulation determines the risk assessment purpose and scope, guiding the gathering of information and data. It presumes the formulation of risk hypotheses, which in turn are shaped by previous experience and knowledge and will be tested against available data (Wolt et al., 2010).

The concepts of familiarity and history of safe use (HOSU) are an integral part of problem formulation, as the availability of existing information is a critical element that adds to the weight of evidence. The Organisation for Economic Co-operation and Development (OECD) was among the first to articulate some of the core principles of familiarity for environmental risk assessment of genetic modified organisms (GMOs) in the Blue Book, back in 1986 (OECD, 1986); and later (OECD, 1993), the basic principles for environmental risk assessment were consolidated and globally accepted to this day. Regarding the food and feed safety assessment of GMOs, the Codex Alimentarius issued specific principles a decade later that constitute the global standard reference (Codex Alimentarius, 2003).

The concept of familiarity involves knowledge and experience that can be used for risk analysis and helps to identify if and what additional knowledge is really needed; therefore, it is not equivalent to safety (Constable et al., 2007).

In September 2018, a workshop facilitated by the International Life Sciences Institutes in Argentina and Brazil discussed the practice of the risk assessment of GMOs in Latin America and identified that the terms “familiarity” and “HOSU” were not clearly defined or were not consolidated as a concept in the literature or guidelines. This group concluded that a consensus would be required on these terms as important tools with harmonization potential for regulatory criteria (ILSI Brasil, 2018). The interpretation and practical implications of the use of these terms by risk assessors in other countries of the Americas were recognized as very relevant. The United States Department of Agriculture (USDA) Animal and Plant Health Inspection Service (APHIS) guideline for “extensions” (USDA-APHIS, 2016), as well as the Canadian approach to similar plants with novel traits (PNTs) (Canada/CFIA, 2018) are excellent examples of how experience with risk assessment and accumulated knowledge can be leveraged to enhance efficiency while keeping a high regulatory standard.

A CAST publication from the same year (Council for Agricultural Science and Technology [CAST], 2018) also addressed familiarity as a key element to reduce the time and effort for decision making and be more efficient in the use of public resources. The following quote gives the flavor of the discussion: “Regulatory agencies…should be prepared to focus questions on identifying new pathways to risk assessment endpoints associated with products that are **unfamiliar** and that require more complex risk assessments.”

Based on these precedents, ILSI Argentina and Brazil held a session at ISBR 15th (April 2019) to elaborate on the familiarity concept and derived tools, and their role in the evolution of risk evaluation criteria. Examples of how different countries in the Americas interpret and apply these conceptual tools were discussed and are presented here.

## Familiarity in the Context of Problem Formulation

Data to support problem formulation can be derived from multiple sources; for the case of transgenic crops, published literature on the biology of the crop, genes, and expression products, and existing documentation on molecular, compositional, and agronomic/phenotypic data are all relevant sources. This information is often available and can help refine and/or reduce the hypotheses that need to be tested for risk characterization (Garcia-Alonso, 2010). In this way, the study plan will include only those tests that must be conducted, as indicated by the problem formulation exercise (Romeis et al., 2009).

By definition, “familiarity” (knowledge and experience) helps in addressing uncertainty in the risk assessment because it is based on preexisting knowledge, experimental evidence, and experience gained over time (OECD, 1993; Hokanson et al., 1999).

Three main knowledge-based factors have driven the evolution of risk assessment criteria for transgenic crops in many parts of the world during the last decade, namely, advances in the knowledge of the intrinsic plasticity of plant genomes (Doebley et al., 2006; Weber et al., 2012;Anderson et al., 2016), of the genomic/genetic effects of transgenesis compared to conventional breeding (Baudo et al., 2006; Batista et al., 2008), and of the natural variability of biochemical composition of the most important crop plants (OECD, 2006, 2002-2012; Ricroch, 2012; Venkatesh et al., 2014; CERA, 2015). This body of knowledge, along with extensive data from the characterization of transgenic events, plus the experience of use of transgenesis in plant breeding, has greatly increased the level of familiarity with the technology (Burachik, 2010; Schnell et al., 2014; Beker et al., 2016).

Experience with the practice of risk assessment is also in itself a substantial component of familiarity, as experienced risk assessors will integrate scientific advances to their own risk assessment experience, contributing to the evolution of evidence-based criteria (USDA-APHIS, 2018).

As for the term “HOSU,” a high level of ambiguity can be found in the language used in guidelines or international documents. According to the OECD, “A long HOSU is a reassuring and practical starting point, for evaluating the safety of a novel food” (OECD, 1999), although “long” is not defined. Similarly, vague language is found in regulatory guidelines: “A substance may be considered to have a HOSU as a food if it has been an ongoing part of the diet for a number of generations in a large, genetically diverse human population where it has been used in ways and at levels that are similar to those expected or intended in Canada” (Health Canada, 2006), or “related, among others, with consumption habits and the massive consumption of the GMO in other countries over years” (Ministerio de Agricultura Ganadería y Pesca, 2013). Specific dates are also found as defining HOSU: Europe defines novel foods as “any food that was not used for human consumption to a significant degree within the Union before 15 May 1997,” or, for traditional foods from third countries, “foods should have been consumed in at least one third country for at least 25 years as a part of the customary diet of a significant number of people” (Engel et al., 2011; EU, 2015).

**FIGURE 1:**
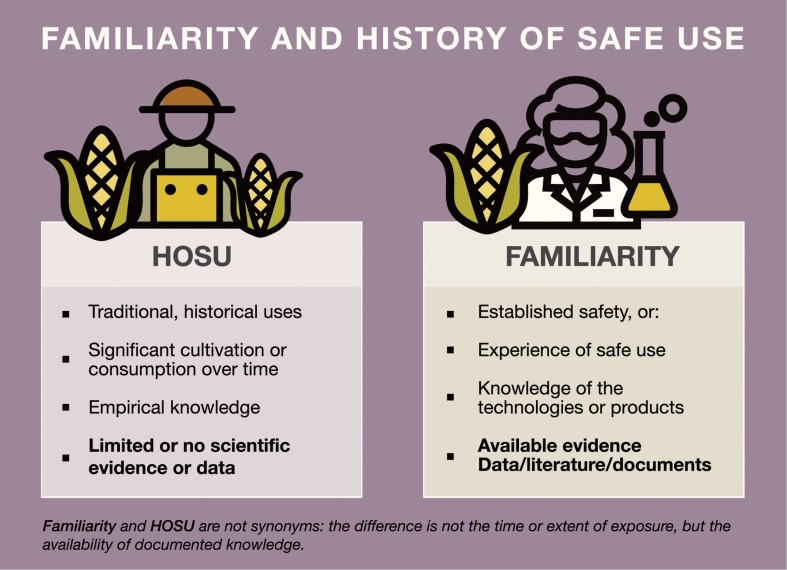
Proposed differences between HOSU and Familiarity.

Although HOSU and familiarity are related concepts, these are not synonymous, even when these terms are frequently used interchangeably. HOSU should be preferably used for traditional uses, of which scientific procedures or formal knowledge would not necessarily be available or may be limited. Familiarity, on the other hand, refers to the body of knowledge (evidence/data) and experience (of use, but also with risk assessment) with technologies and products that have undergone a risk assessment process or for which substantial data are available (Figure 1). Ambiguous language can create confusion and ultimately leads to discretionary interpretations and less predictable risk assessment processes (Wasmer, 2019). To exemplify the relevance of clear definitions, in the specific case of transgenic comparators for field studies, using HOSU as an acceptance criterion would be discretional. Familiarity, on the other hand, would describe the availability of documented knowledge that would allow for using these, as well as null segregants as suitable comparators.

Clear and consistent definitions enhance transparency and facilitate conceptual harmonization for modern, evidence-based risk assessments.

## The Use of Familiarity and a History of Safe Use in the Decisions of the Brazilian National Biosafety Technical Commission

The current GMO legislation in Brazil centers around the Biosafety Law and Decree (BRASIL, 2005), and Norms and Technical decisions^1^ issued by the National Biosafety Technical Commission (CTNBio).

The heart of the Brazilian GMO Biosafety policy is CTNBio^2^, a consulting and deliberating multidisciplinary collegiate body that formulates the norms, examines the evidence, and authorizes any activity related to GMOs.

Even though neither the Biosafety Law and Decree nor CTNBio’s Normative Resolutions mention the terms “familiarity” or “HOSU” in the context of risk assessment of GMOs and their by-products, these concepts are implicit in the assessments performed by its members. As experienced scientists (all members of CTNBio must hold a doctorate degree, have acknowledged technical competence, and should have been professionally active in the biosafety, biotechnology, biology, human or animal health areas, and the environment), the use of the scientific method is an intrinsic part of their analysis.

In fact, observing the many review processes held for the commercial release of GMOs in the last 20 years^3^, we note the use of the terms “history of use,” “safe use,” “safe consumption,” “safe history,” and “HOSU” in several documents. However, the term “familiarity” is not used. We believe that this is due to a lack of a standard definition and therefore of a misconception of the term. CTNBio’s risk assessors, in writing their technical opinions, infer that there is knowledge (evidence/data) and experience in the use of technologies and products, in particular, those who have undergone a risk assessment process or for which substantial data are available, in other words, familiarity.

The Brazilian Biosafety Law establishes that all activities related to GMOs in the country must be “guided by the drive for attaining scientific development in the biosafety and biotechnology area, the protection of life and human beings, of animal and plant health, and the compliance with the principle of environmental precaution.” In addition, it is the responsibility of the proponent to establish that the proposed activity will not (or is very unlikely to) result in significant harm.

Normative Resolution No. 05 of CTNBio (BRASIL, 2007) mentions “the history of use for food and feed of the GMO unmodified parent” and “the history of cultivation and usage of the GMO unmodified parent in the environment” as key pieces of information to consider. In addition, CTNBio Technical Decisions have consistently reflected (even with no mention in the law) the application of conceptual tools based on familiarity, as data for human and animal health risk assessment performed in other parts of the world are considered. However, as established in the same normative, environmental evidence for risk assessment has to be generated in the ecosystems in which the particular plant will be cultivated.

National Biosafety Technical Commission has evaluated and approved four yeast strains for the production of first- and second-generation ethanol, three yeast strains, and seven microalgae for oil production, in addition to a large quantity of animal vaccines, until 2018. Recently, four varieties of GM corn have been approved for marketing exclusively for human and animal consumption, although they cannot be grown in Brazil because they have not been tested in the Brazilian edaphoclimatic conditions as required; however, these assessments did consider available information generated elsewhere, and therefore, the concept of familiarity was used.

Finally, as stated in the Brazilian Biosafety Law: “CTNBio shall monitor the development and technical-scientific progress attained by the biosafety, biotechnology, bioethics and related areas, with aims at increasing their capacity of protecting human, animal and plant health and the environment.” This provision legally ensures that CTNBio’s decisions are based on the most current scientific knowledge and state of the art.

## The Regulation of Agricultural Biotechnology and Science: A Canadian Perspective on the Conceptual Tools for Problem Formulation

During the scientific consultations carried out in the late 1980s on biotechnology-derived plants, it was agreed that the regulatory scope should be focused on plants with traits sufficiently different from those already present in the species, as to require a risk assessment. This led to the recommendation that the product and not the process would be regulated, and the scientific perspective that came from these consultations was that plants derived through genetic engineering were not necessarily any riskier than those derived through chemical mutagenesis or other breeding techniques. This resulted in a regulatory approach that created the basis for the effective incorporation of science into policy, giving rise to the articulation of the 1993 Federal Regulatory Framework for Biotechnology^4^. This framework described an approach to biotechnology, based on the use of science-based safety assessments and risk management, aimed at protecting human and animal health, and the environment and at the same time providing an environment that allowed for innovation. All current regulatory frameworks for transgenic plants incorporate the need for a risk assessment prior to environmental release, to identify and evaluate the risks associated with the release and cultivation of these plants using a comparative approach.

Key to the environmental risk assessment is a thorough knowledge of the crop species that has been subject to modification by biotechnology to express a new trait. This knowledge is fundamental to conducting a comparative risk assessment. The concept of familiarity is used to identify and evaluate environmental risks that may be associated with the release of a transgenic plant and to inform management practices that may be needed to mitigate recognized risks. In Canada, this requirement is satisfied by the creation of individual-crop biology documents. These documents describe the behavior of the crop species specifically in the Canadian environment and include a description of relevant parameters (plant growth, reproduction, interactions with related and unrelated species, management practices, etc.) to inform the risk assessment (CFIA, 2017). Although similar in focus to the consensus documents developed by the OECD (2006), the Canadian document describes management conditions and environmental interactions for the unmodified species that are specific to the Canadian environment, and uses the familiarity with the cultivation and management of a species as the basis to identify potential hazards during the safety assessment.

Using familiarity as a guiding principle and considering pathways to harm, a hypothesis that growing a certain GM crop will cause no harm is really a hypothesis that growing the GM crop will cause no greater harm than that cultivation of the non-GM crop it may replace. For the risk assessment, “a hypothesis that growing a certain GM crop will pose no unacceptable risk is really a hypothesis that any increase in risk caused by growing the GM crop will be acceptable” (Raybould and Macdonald, 2018).

The principles of the comparative risk assessment, the use of familiarity, and the Canadian product-based approach (CFIA, 2017) were evident in a recent incident when Canadian regulators, like those in other countries, became aware that petunias that had been genetically engineered to produce orange flowers by using a gene from corn were potentially present in Canada. Regulators in Canada considered relevant information and scientific rationale, and determined that the GM petunias pose no more risk to the environment than conventional petunias, and in line with the product-based approach, they would not be regulated in Canada. Since there was no scientific evidence that the GM petunias posed any risk to the environment, distributors or producers of the GM petunias were not required to remove them from the supply chain.

For the crops we know well, the concept of familiarity and the comparative risk assessment approach has provided a useful paradigm for risk assessments. In fact, today most of the maize, soybeans, and canola grown by Canadian farmers are a product of biotechnology. As techniques such as gene editing push more new varieties forward to the marketplace, these sound principles for risk assessment, anchored in a strong policy framework, will allow Canadian farmers safe access to these new varieties.

## Conceptual Tools Based on Familiarity. Transportability of Field Studies From Brazil to Argentina: A Case Study

The conceptual framework for data transportability (DT) builds on the premise that results from well-designed studies conducted for the environmental and food/feed risk assessment of transgenic crops may be relevant and therefore transportable to other geographies (Garcia-Alonso et al., 2013). This concept focuses not only on methodological quality but also on the familiarity with crops, traits, and receiving environments.

Bean crop (*Phaseolus vulgaris*) production took relevance in Argentina in the 70s as an alternative for rotation with other crops. One of the main diseases causing important yield losses is golden mosaic, caused by the *Bean Golden mosaic virus* (BGMV). In 2011, a transgenic bean resistant to BGMV was approved in Brazil for cultivation and consumption, developed by EMBRAPA (*Brazilian Agricultural Research Company*), through an RNA interference mechanism (BRASIL, 2011). ILSI Argentina’s Biotechnology Working Group was interested in testing the applicability of the framework to a real case and, to this end, convened a subteam to discuss this particular case as an example.

A set of regulatory field studies carried out by EMBRAPA in Brazil were reviewed to discuss their transportability to the argentine receiving environment. This discussion considered that information generated in field trials is transportable, provided that trials are properly designed and conducted in diverse agroclimatic environments, allowing for the expression of any biologically relevant phenotypic differences. Under these considerations, sites selection, methodologies, and agronomic management of the studies were examined with focus on protocol and end point consistency, record keeping, and traceability. Familiarity with the crop and the bean cultivation zones in Argentina was also considered. The group concluded that the trials were transportable from Brazil to Argentina and might be eventually applicable to a risk evaluation process, provided that assessment end points would respond to the risk hypotheses identified according to regulatory requirements (Vesprini et al., 2019).

## Conclusion

The concepts of familiarity and HOSU are an integral part of problem formulation. Although related concepts, they are not synonymous, in spite of the fact that they are often used interchangeably. Ambiguous language leads to discretionary interpretations and less predictable risk assessment processes. Clear and consistent definitions are needed to enhance transparency and facilitate conceptual harmonization for modern, evidence-based risk assessments.

This document intents to highlight the need for clearer definitions of these terms for the case of transgenic crops and propose to differentiate both terms based on the availability of documented knowledge. In this way, *Familiarity* should refer to the body of knowledge and experience with technologies and products that have undergone a risk assessment process or for which substantial data are available. *HOSU*, on the other hand, should be preferably used for traditional uses, of which scientific procedures or formal knowledge would not necessarily be available or may be limited.

The continued commitment in the practice of risk assessment of those who have direct responsibility for regulatory oversight leads to the integration of scientific advances in their own risk assessment experience, thus contributing to the evolution of evidence-based criteria. In other words, it allows for integrating familiarity into regulatory decisions. Collaboration among regulatory agencies is essential to this end.

## Author Contributions

All authors participated in the drafting of this manuscript as individual experts in their fields, and the authors are solely responsible for the contents. Any views expressed in this manuscript are the views of the authors and do not necessarily represent the views of any organization, institution, or government with which they are affiliated or employed.

## Conflict of Interest

The authors declared their affiliations and employers in the authors list. CR was employed by the company Bayer Crop Science Argentina. The remaining authors declare that the research was conducted in the absence of any commercial or financial relationships that could be construed as a potential conflict of interest.

## Footnotes

1 Available at: http://ctnbio.mcti.gov.br/resolucoes-normativas/

2 Available at: http://ctnbio.mcti.gov.br/inicio

3 Available at: http://ctnbio.mcti.gov.br/en/liberacao-comercial#/liberacao-comercial/consultar-processo

4 Available at: https://www.ourcommons.ca/Content/Archives/Committee/352/sust/reports/03_1996-11/chap2-e.html

## References

[B1] Anderson J. E. Michno J. M. Kono T. J. Y. Stec A. O. Campbell B. W. Curtin S. J. (2016). Genomic variation and DNA repair associated with soybean transgenesis: a comparison to cultivars and mutagenized plants. *BMC Biotechnol.* 16:41. 10.1186/s12896-016-0271-z 27176220 PMC4866027

[B2] Batista R. Saibo N. Lourenco T. Oliveira M. M. (2008). Microarray analyses reveal that plant mutagenesis may induce more transcriptomic changes than transgene insertion. *Proc. Natl. Acad. Sci. U.S.A.* 105 3640–3645. 10.1073/pnas.0707881105 18303117 PMC2265136

[B3] Baudo M. M. Lyons R. Powers S. Pastori G. Edwuards K. Holdsworth M. (2006). Transgenesis has less impact on the transcriptome of wheat grain than conventional breeding. *Plant Biotechnol. J.* 4 369–380. 10.1111/j.1467-7652.2006.00193.x 17177803

[B4] Beker M. P. Boari P. Burachik M. Cuadrado V. Junco M. Lede S. (2016). Development of a construct-based risk assessment framework for genetic engineered crops. *Transgenic Res.* 25 597–607. 10.1007/s11248-016-9955-3 27339146 PMC5023744

[B5] Borges B. J. P. Arantes O. M. N. Fernandes A. A. R. Broach J. R. Fernandes P. M. B. (2018). Genetically modified labeling policies: moving forward or backward? *Front. Bioeng. Biotechnol*. 6:181. 10.3389/fbioe.2018.00181 30538985 PMC6277523

[B6] BRASIL (2005). *Law 11.105, March 24, 2005. Diário Oficial [da] República Federativa do Brasil, Brasília* Available at: http://www.planalto.gov.br/ccivil_03/_Ato2004-2006/2005/Decreto/D5591.htm (accessed March 28, 2005).

[B7] BRASIL (2007). *Ministério da Ciência e Tecnologia. Comissão Técnica Nacional de Biossegurança. Resolução Normativa CTNBio Nº 4.* Available at: http://ctnbio.mcti.gov.br/en/resolucoes-normativas/-/asset_publisher/OgW431Rs9dQ6/content/resolucao-normativa-n%C2%BA-4-de-16-de-agosto-de-2007?redirect=http%3A%2F%2Fctnbio.mcti.gov.br%2Fen%2Fresolucoes-normativas%3Fp_p_id%3D101_INSTANCE_OgW431Rs9dQ6%26p_p_lifecycle%3D0%26p_p_state%3Dnormal%26p_p_mode%3Dview%26p_p_col_id%3Dcolumn-2%26p_p_col_count%3D3%26_101_INSTANCE_OgW431Rs9dQ6_advancedSearch%3Dfalse%26_101_INSTANCE_OgW431Rs9dQ6_keywords%3D%26_101_INSTANCE_OgW431Rs9dQ6_delta%3D15%26p_r_p_564233524_resetCur%3Dfalse%26_101_INSTANCE_OgW431Rs9dQ6_cur%3D2%26_101_INSTANCE_OgW431Rs9dQ6_andOperator%3Dtrue (accessed August 16, 2007).

[B8] BRASIL (2011). *Parecer Técnico nº 3024.2011.* Available at: http://ctnbio.mcti.gov.br/liberacao-comercial;jsessionid=062C1E657E86449B431548AF46081760.columba?p_p_id=110_INSTANCE_SqhWdohU4BvU&p_p_lifecycle=0&p_p_state=normal&p_p_mode=view&p_p_col_id=column-2&p_p_col_count=3&_110_INSTANCE_SqhWdohU4BvU_struts_action=%2Fdocument_library_display%2Fview_file_entry&_110_INSTANCE_SqhWdohU4BvU_redirect=http%3A%2F%2Fctnbio.mcti.gov.br%2Fliberacao-comercial%2F-%2Fdocument_library_display%2FSqhWdohU4BvU%2Fview%2F686135%3Bjsessionid%3D062C1E657E86449B431548AF46081760.columba&_110_INSTANCE_SqhWdohU4BvU_fileEntryId=686151#/liberacao-comercial/consultar-processo (accessed April 5, 2018).

[B9] Burachik M. (2010). Experience from use of GMOs in Argentinian agriculture, economy and environment. *N. Biotechnol.* 27 588–592. 10.1016/j.nbt.2010.05.011 20580682

[B10] CERA (2015). *GM Crop Database. Center for Environmental Risk Assessment (CERA).* Washington, DC: ILSI Research Foundation.

[B11] CFIA (2017). *Directive 94-08 (Dir 94-08) Assessment Criteria for Determining Environmental Safety of Plants With Novel Traits.* Available at: https://www.inspection.gc.ca/plants/plants-with-novel-traits/applicants/directive-94-08/eng/1512588596097/1512588596818 (accessed May 19, 2019).

[B12] CFIA (2018). *Directive 94-08 (Dir 94-08) Revised Assessment Criteria for Determining Environmental Safety of Plants With Novel Traits.* Available at: https://www.inspection.gc.ca/plants/plants-with-novel-traits/applicants/directive-94-08/appendices/eng/1512662253920/1512662254595#app5 (accessed May 19, 2019).

[B13] Codex Alimentarius (2003). *Principles for the Risk Analysis of Foods Derived From Modern Biotechnology. CAC/GL 44-2003.* Rome, Italy: Joint FAO/WHO Food Standards Programme, 4.

[B14] Constable A. Jonas D. Cockburn A. Davi A. Edwards G. Hepburn P. (2007). History of safe use as applied to the safety assessment of novel foods and foods derived from genetically modified organisms. *Food Chem. Toxicol.* 45 2513–2525. 10.1016/j.fct.2007.05.028 17692450

[B15] Council for Agricultural Science and Technology [CAST] (2018). *Genome Editing in Agriculture: Methods, Applications, and Governance—A Paper in the Series on the Need for Agricultural Innovation to Sustainably Feed the World by 2050. Issue Paper* 60. Ames, IA: Council for Agricultural Science and Technology.

[B16] Doebley J. F. Gaut B. S. Smith B. D. (2006). The molecular genetics of crop domestication. *Cell* 127 1309–1321. 10.1111/j.1439-0418.2009.01423.x 17190597

[B17] Engel K.-H. Vogel R. F. Knorr D. Habermeyer M. Kochte-Clemens B. Eisenbrand G. (2011). The role of the concept of “history of safe use” in the safety assessment of novel foods and novel food ingredients. Opinion of the senate commission on food safety (SKLM) of the German research foundation (DFG). *Mol. Nutr. Food Res.* 55 957–963. 10.1002/mnfr.201100206 21538858

[B18] EPA (2014). *Framework for Ecological Risk Assessment.* Washington, DC: U.S. Environmental Protection Agency. 10.1002/mnfr.201100206

[B19] EU (2015). *New Novel Foods Directive.* Available at: https://eur-lex.europa.eu/legal-content/EN/TXT/?uri=CELEX:32015R2283 (accessed April 5, 2019).

[B20] Garcia-Alonso M. (2010). Current challenges in environmental risk assessment: the assessment of unintended effects of GM crops on non-target organisms. *IOBC/WPRS Bull.* 52 57–63.

[B21] Garcia-Alonso M. Hendley P. Bigler F. Mayeregger E. Parker R. Rubinstein C. (2013). Transportability of confined field trial data for environmental risk assessment of genetically engineered plants: a conceptual framework. *Transgenic Res.* 23 1025–1041. 10.1007/s11248-014-9785-0 24733670 PMC4204004

[B22] Health Canada (2006). *Guidelines for the Safety Assessment of Novel Foods Food Directorate Health Products and Food Branch Health Canada June, 2006.* Available at: https://www.canada.ca/content/dam/hc-sc/migration/hc-sc/fn-an/alt_formats/hpfb-dgpsa/pdf/gmf-agm/guidelines-lignesdirectrices-eng.pdf (accessed May 19, 2019).

[B23] Hokanson K. Heron D. Gupta S. Koehler S. Roseland C. Shantharam S. (1999). *The Concept of Familiarity and Pest Resistant Plants.* Lincoln, NE: USDA-ARS/UNL Faculty, 479.

[B24] ILSI Brasil (2018). *Conceito de Familiaridade na Avaliação do Risco: Experiência das Américas.* Available at: https://ilsibrasil.org/event/conceito-de-familiaridade-na-avaliacao-do-risco-experiencia-das-americas/ (accessed December 3, 2018).

[B25] Ministerio de Agricultura, Ganadería y Pesca (2013). New Regulatory Framework for Agrobiotechnology in Argentina (Nuevo Marco Regulatorio para la Biotecnologia Agropecuaria en la Argentina). Available at: https://www.argentina.gob.ar/agricultura/alimentos-y-bioeconomia/marco-regulatorio (accessed April 5, 2019).

[B26] Norton S. Rodier D. Van der Schalie W. Wood W. Slimak M. Gentile J. (1992). A framework for ecological risk assessment at the EPA. *Environ. Toxicol. Chem. Annu. Rev.* 11 1663–1672.

[B27] OECD (1986). *Recombinant DNA Safety Considerations. Safety Considerations for Industrial, Agricultural and Environmental Applications of Organisms Derived by Recombinant DNA Techniques (“The Blue Book”).* Paris: Organisation for Economic Co-operation and Development.

[B28] OECD (1993). *Safety Considerations for Biotechnology: Scale-up Considerations of Crop Plants.* Paris: Organization for Economic Co-operation and Development.

[B29] OECD (1999). *GM Food, Regulation and Consumer Trust. OECD Observer No. 216.* Paris: Organisation for Economic Co-operation and Development, 21.

[B30] OECD (2006). *Safety Assessment of Transgenic Organisms: OECD Consensus Documents, Vol. 1, Biology of Crops.* Paris: Organization for Economic Co-operation and Development

[B31] OECD (2002-2012) *Consensus Documents for the Work on the Safety of Novel Foods and Feeds: Compositional Considerations.* Paris: Organization for Economic Co-operation and Development

[B32] Patton D. E. (1998). Environmental risk assessment: tasks and obligations. *Hum. Ecol. Risk Assess.* 4 657–670. 10.1080/10807039891284532

[B33] Raybould A. Macdonald P. (2018). Policy-led comparative environmental risk assessment of genetically modified crops: testing for increased risk rather than profiling phenotypes leads to predictable and transparent decision-making. *Front. Bioeng. Biotechnol.* 6:43. 10.3389/fbioe.2018.00043 29755975 PMC5932390

[B34] Ricroch A. (2012). Assessment of GE food safety using ‘-omics’ techniques and long-term animal feeding studies. *N. Biotechnol.* 30 349–354. 10.1016/j.nbt.2012.12.001 23253614

[B35] Romeis J. Lawo L. C. Raybould A. (2009). Making effective use of existing data for case-by-case risk assessments of genetically engineered crops. *Appl. Entomol.* 133 571–583. 10.1111/j.1439-0418.2009.01423.x

[B36] Schnell J. Steele M. Bean J. Neuspiel M. Girard C. Dormann N. (2014). A comparative analysis of insertional effects in genetically engineered plants: considerations for pre-market assessments. *Transgenic Res.* 24 1–17. 10.1007/s11248-014-9843-7 25344849 PMC4274372

[B37] USDA-APHIS (2016). *Guidance on Petitions for Extensions of Non-Regulated Status.* Available at: https://www.aphis.usda.gov/aphis/ourfocus/biotechnology/sa_permits_notifications_and_petitions/sa_guidance_documents/ct_extensions (accessed April 29, 2019).

[B38] USDA-APHIS (2018). *Notice of Intent to Prepare an Environmental Impact Statement; Movement and Outdoor Use of Certain Genetically Engineered Organisms.* Available at: https://www.aphis.usda.gov/brs/fedregister/BRS_20180629.pdf

[B39] Venkatesh T. V. Cook K. Liu B. Perez T. Willse A. Tichich R. (2014). Compositional differences between near-isogenic GM and conventional maize hybrids are associated with backcrossing practices in conventional breeding. *Plant Biotechnol. J.* 13 200–210. 10.1111/pbi.12248 25196222

[B40] Vesprini F. Maggi A. López Olaciregui M. Módena N. Seipel M. (2019). “Case study: transportability of virus resistant transgenic common bean- field studies from Brazil to Argentina,” in *Proceedings of the 15th ISBR Symposium Tarragona, Spain. Parallel Session 3: Familiarity in the Context of Risk assessment of Transgenic Crops in the Americas, Organizers*, eds Capalbo C. Vicién C. (Buenos Aires: Biotechnology Directorate of the Secretary of Agroindustry).

[B41] Wasmer M. (2019). Roads forward for European GMO Policy—Uncertainties in wake of ECJ judgment have to be mitigated by regulatory reform. *Front. Bioeng. Biotechnol.* 7:132. 10.3389/fbioe.2019.00132 31231643 PMC6561310

[B42] Weber N. Halpin C. Hannah C. L. Jez J. M. Kough J. Parrott W. (2012). Crop genome plasticity and its relevance to food and feed safety of genetically engineered breeding stacks. *Plant Physiol.* 160 1842–1853. 10.1104/pp.112.204271 23060369 PMC3510115

[B43] Wolt J. Keese P. Raybould A. Fitzpatrick J. Burachik M. Gray A. (2010). Problem formulation in the environmental risk assessment for genetically modified plants. *Transgenic Res*. 19 425–436. 10.1007/s11248-009-9321-9 19757133 PMC2865628

